# Meeting September 6

**Published:** 1886-10

**Authors:** 


CHICAGO MEDICAL SOCIETY.

*Stated Meeting, September 6, 1886.*—E. J. DOERING, M. D., President, in the chair.

PROFESSOR J. S. JEWELL gave a partial verbal abstract of his paper on

OVERFILLING AND DILATATION OF THE COLON.

He said that for the last ten years this subject had attracted more or less of his attention. During the last eight or ten years he had accumulated a list of over five hundred carefully studied cases, in which the condition named existed and concerning which he gave his observations. His mode of investigating the cases in which the overfilling existed has been as follows: The patient is stripped so that the abdomen is bare, the abdominal walls being relaxed. Then he takes a plate of soft rubber at least two inches in length by an inch and a half in breadth and one quarter inch in thickness, and a heavy steel hammer, quite as heavy as the ordinary surgical bone hammer. With this the abdomen is carefully percussed over the track of the colon, from its origin, tracing it up under the liver, across and down to the sigmoid flexure. Thus percussing over the track of the colon, and carefully locating every mass of fæces, even in persons with fleshy abdominal walls, he has been able to get at its course and contents. Out of these five hundred cases, perhaps a hundred have said that they were as "regular as clockwork" in having movements from their bowels. But upon examination, in most of such cases the colon was found to have variable quantities of fæcal material lying in it. The part of the colon most subject to dilatation is the sigmoid flexure and the descending colon. At the flexure of the colon, deep in the left hypochondriac region, almost as frequent as in any other part of the course of the colon, he had found it dilated and filled with fæcal matter. The next most common situation is the transverse colon, last of all the cæcum. In one case where the colon was very largely dilated, during the thirty or forty hours that were devoted to emptying it, not continuously but at different times, two gallons or more of fæces were removed. The usual condition is to find masses of fæces lying in lumps here and there, but not filling the entire intestine.

In one case a mass was removed from the right extremity of the colon by massage, and flooding the colon its entire length with water. The fact that this mass had remained in the colon for more than nine months was ascertained in this way: The patient had taken a dish of dewberries, such as appear in the fall after the ordinary blackberry has passed its season. He was in ill health, and growing worse during his visit he returned home. This was in September. Professor Jewell saw him early in June following. When the mass was removed it was found to be more or less filled with the seeds of the dewberry, and according to his own account he had not taken berries of any kind since the September previous, certainly no dewberries. This mass was about the consistence of soft putty and the size of a large Irish potato.

In most of the cases investigated the colon was dilated apparently, in some part or other, all the way from two to three times its proper calibre. This class of patients are all liable to become "bilious," and are habitually taking cathartic pills, mineral waters, etc. In all or nearly all instances Professor Jewell could trace their "biliousness" to gradual filling up of the colon, and the disturbance produced from this cause in the digestive apparatus higher up. The change in the secretions of the mouth, the bad breath, the unhealthy odor of the skin produced by absorption of fœtid substances from the rotting masses in the intestine, together with the coloring matter in the same, gives the skin of the patient a peculiar, dirty, sallow color. One of the first things to occur in addition to the dilatation, and weakening of the muscular coat of the colon by accumulation of material in it, is irritation of portions of the mucous membranes where the fœcal masses lie, producing local catarrhs. This occurs with a frequency and to an extent that no one would suspect unless the patient or the nurse should carefully watch the stool. The next important factor in these cases is irritation of the peripheral ends of the sensory nerves of the intestinal mucous membrane, irritated by contact with the fœcal masses. The nerves that return from the colon probably enter the spinal cord at a level not lower than that of the tenth or eleventh dorsal vertebra. Above the level named they probably enter the cord at various points higher up in the lower two-thirds of the dorsal cord, not to mention direct medulla connections, etc. Along these nerves are propagated the irritative impressions from the colon into the spinal cord or medulla, and thereby "switch" connections outgoing or reflex impulses are sent to other portions of the body, producing thus various forms of disorder to be referred to later. He believes that he has not found one case out of twenty of ordinary melancholia that has not had its origin partly or in some cases apparently entirely in overfilling of the colon. Headache and a great many transient disorders referred to the head more frequently owe their origin to this source than he had ever supposed. Next is the influence upon the circulation in general, especially upon the heart's action, lowering its tension, and thus giving a feeble pulse.

Dilatation of the colon often causes a sluggish capillary circulation, and this accounts for the frequency with which patients complain of cold extremities. Professor Jewell has found patients who could not remember having had naturally warm feet for years, when upon removal of the masses in the colon the feet would become warm. In insomnia he has found the condition of the colon described to be a frequent cause, the colon being the seat of the original disorder. There is lowering of the vascular tension nearly always in these cases, hence the feeble nutrition and feeble circulation in the brain, not to mention other parts. The next most important result of dilatation and overfilling of the colon is the fact that these masses of retained fæcal substance in various ways poison the individual by absorption of septic material. He has had three cases of epilepsy that he traced exclusively, as far as could be told by the results of treatment afterwards, to disease in the left extremity of the colon caused by fæcal accumulations, and when the disorder was removed by proper means he was able to relieve the epilepsy. For the removal of these masses, as a rule, he does not use purgatives, but injections are given after the following plan: A large bag fountain syringe, holding half a gallon, is adjusted at a convenient height from the floor, the patient lying down in the knee-chest position. The fluid should flow with considerable force until it makes its way past the upper sphincter which is at the lower part of the sigmoid flexure; pass one quart to one-half gallon of water; according to the size of the colon, so as to fill the entire organ to the cæcum. In some instances he has been able to introduce as much as a gallon of water into the colon, the patient feeling no serious inconvenience from it, and a half gallon is a common amount to introduce. The patient is then directed to lie down on the left side, and by proper massage over the ascending colon the liquid is moved and with it the contents of the bowel toward the rectum. This should be done once or twice in the twenty-four hours until the colon is clear, and by this means it should be kept so. After this has been accomplished give the patient something that will act as a stimulant to the nervo-muscular coat of the colon. A good prescription for this purpose he has found to be, either in pill or capsule form, extract of cascara, a little belladonna, strychnia, hydrastis canadensis and aloes. By care the colon can be kept clear, and the disorders of the stomach usually disappear, the secretions of the mouth are improved, the skin becomes of a healthier color and loses the distinctly fæcal odor that it frequently has.

He has met with a number of cases of so-called typho-malarial fever in their earlier stage with a temperature as high at times as 103–4–5, in which, by removing a great mass of material undergoing rapid fermentative decomposition at the high heat of the body, leading to rapid distension of the colon with gas, the signs of disease abated in a few hours and rapid recovery occurred. Where there is considerable inflammation in the bowel itself he introduces water pretty well charged with listerine or some other antiseptic, and a little glycerine or other medicinal agents, and in this way the intestine is purified and cleansed and the individual immediately improved. It seems now astonishing that it never occurred to him earlier, that to have a mass of fæces undergoing fermentation and decomposition in the interior of the body, and in contact with an absorbent surface like that of the mucous membrane of the colon is, or often may be, the real cause of serious diseases.

PROFESSOR H. A. JOHNSON in opening the discussion said: This is a subject to which he had given no special attention, but perhaps if he should propose one or two questions they might elicit some information. First, the inquiry suggests itself as to why this state of things exists, what is the cause of it? Perhaps as the author has suggested, our rapid mode of eating, and our neglect of the natural demands of the functions of digestion have something to do with it, but this does not seem to cover it all. Secondly, the question arises as to how far such a state of things as he describes does actually exist in the colon, viz., putrefaction of the contents. So far as his observation goes, the contents of the cavities of the body, including the alimentary canal and bladder, are kept from undergoing these putrefactive changes while they are inclosed. It is true that the fæcal matters are full of bacteria in various forms, but he is not sure that the ordinary processes that take place out of the body, go on here. Thirdly, as to the function of the colon; it is both an excretory and secretory organ, and nutritive matter that is carried into it is undoubtedly taken up in its passage through it. There should be a delay, a normal arrest of the progress of the fæcal mass from the time it enters the colon at the cæcum till its discharge. In the normal condition the fæcal mass is made up of the debris, of the waste matters of food, and the material removed from the blood; there ought to be, physiologically, a retardation—time for the accomplishment of these two functions. What is that time?

DR. WM. E. CLARK said: The subject is one of great importance, and one to which he had given much thought. His first call in consultation, after his return to the city some twenty years ago, was to a female patient who had been an invalid for a number of years. A tumor could be felt through the abdominal walls, supposed by the attending physician to be a tubercular enlargement of the mesenteric glands, but which proved to be a mass of fæcal matter in the colon. It had probably been accumulating for a long time, as after removal it was found largely to consist of bran from Graham bread—her principal diet for several years previous. Under appropriate treatment, with particular attention to the condition of her bowels, the patient apparently recovered her health, though she died some years after with disease of the kidneys—the latter perhaps having some relation to the previous condition of the bowels.

He had just made an examination of a supposed uterine tumor, and found a tumor but not connected with the uterus

—but an accumulation of fæcal matter in the colon, and consequently made arrangements for its evacuation. For the removal of these masses of fæcal matter, he first gave cathartic with large doses of olive oil and then used injections of water into the bowel as far up as possible through a stomach-tube; sometimes resorting to the placental forceps for the removal of large fragments.

PROFESSOR G. C. PAOLI said: He had listened with a great deal of interest to the report of Professor Jewell. We know that expansion of the colon occurs in many different diseases, and there is hardly a practitioner who has not seen it in his practice for years. We see it in typhoid fever, nephritis, hysteria and lead-colic. Constipation is undoubtedly a great cause of expansion of the colon. A man gets constipated, neglects himself, his fæces become very hard, and in consequence pains occur in the colon. With due respect to the author's experience and observation, he must admit that although we meet with cases in our practice they are not so common that we see them in the hospital. In regard to the treatment, we ought to administer such remedies as will give tonicity to the muscular coat of the bowels, such as cascara sagrada in combination with small doses of aloes. But without physical exercise medicine has very little beneficial effect. He has seen a shower bath over the abdomen have a good effect in producing contraction and tonicity of the muscular coat of the bowels. In regard to the injection which the author advises, it is well to remark that the rectum should first have a digital examination, because often hard excrement is impacted in it and under such circumstances the injection would meet with an obstacle. Strychnia may be very good in chronic but not in acute cases.

PROFESSOR JEWELL, in closing the discussion, said: The members of the society will of course understand that he had spoken only of the sick people who had come to him, a special class that is not met with so frequently in general practice. He was speaking entirely within bounds when he said that the number of cases that he now has on record in which over-filling of the colon was one of the principal features of disorder, must be 700 or 800 during the last ten years, and 500 of these have been made the basis of his study. The question as to how long the matter may be left in the large intestine without harm is an exceedingly interesting one, and he has made that a study. The difference between the ordinary slow decomposition of fæcal matter in the intestine and unhealthy decomposition has been taken into account in that study, as well as the conditions or causes that bring about unhealthy decomposition. He is convinced that not enough stress is laid upon this subject. Many persons in the condition referred to have been all but ruined by taking by the week, month and year, cathartics, which must be taken into the stomach first, dissolved and go the entire length of the small intestine before they reach the colon. The colon may be emptied in a much more satisfactory way by means of large injections, together with the use of medicinal agents, to stimulate naturally its weakened muscular apparatus.

DR. A. SCHIRMER reported a case of

ACTINOMYCOSIS HOMINIS, with exhibition of patient and specimens of actinomyces. The paper is given in full in another part of the JOURNAL AND EXAMINER.

DR. NEWKIRK asked if the patient knew of any disease among the cattle he attended.

DR. SCHIRMER: He said there was no disease among the cattle, and nobody else had such a disease.

PROFESSOR W. T. BELFIELD said: The case is hardly one for discussion, because Dr. Schirmer has not said anything about actinomycoses as such, but merely about the case. The finding of actinomyces in the sputum is very interesting; it is undoubtedly a case of actinomycosis of the lungs and must prove fatal. There are only some thirty cases recorded in which this parasite has been found in men. He said he was not familiar with the literature on the subject for the last year, but up to that time there had been no well authenticated case of actinomycosis observed in man either in England or America.

DR. A. V. PARK read a paper on a case of

CARIES OF THE RIGHT PARIETAL BONE CAUSED BY RAILWAY INJURY, which will appear in the next number of the JOURNAL AND EXAMINER.

